# EEG‐Based Deep Learning Model for Hyper‐Acute Large Vessel Occlusion Stroke Detection in Mice

**DOI:** 10.1111/cns.70592

**Published:** 2025-09-27

**Authors:** Tan Zhang, Xiaolin Li, Xinxin Hu, Zhiyong Zhou, Qingchun Mu, Xiaoke Chai, Qing Lan, Jizong Zhao

**Affiliations:** ^1^ Department of Neurosurgery The Second Affiliated Hospital of Soochow University Suzhou China; ^2^ Suzhou Institute of Biomedical Engineering and Technology Chinese Academy of Sciences Suzhou China; ^3^ Changchun University of Science and Technology Changchun China; ^4^ Department of Neurosurgery, Beijing Tiantan Hospital Capital Medical University Beijing China

**Keywords:** acute ischemic stroke, deep learning, EEG, *EEGNet*, large vessel occlusion

## Abstract

**Objective:**

This study aims to develop a deep learning model for the early and accurate detection of hyper‐acute large vessel occlusion (LVO) stroke using EEG data.

**Methods:**

A pMCAO mouse model was used to simulate LVO stroke, with high‐resolution EEG data collected during the hyper‐acute phase. *EEGNet*, a specialized deep learning architecture, was employed to develop a model based on EEG signals for the detection of hyper‐acute LVO strokes. Seven‐fold cross‐validation was conducted to evaluate the model's performance across multiple metrics, including accuracy, AUC, precision, recall, and F1 score.

**Results:**

The model achieved an overall accuracy of 97.9% and an AUC of 0.977, demonstrating excellent diagnostic performance across the hyper‐acute phase. Stroke detection was reliable within 1.5 h post‐onset, with classification accuracies exceeding 95% in all five time intervals segmented by hour. t‐SNE analysis confirmed effective feature extraction, and comparisons with sham‐operated mice validated the model's specificity for stroke‐related EEG changes.

**Conclusion:**

The EEG‐based deep learning model showed robust performance in hyper‐acute LVO stroke detection, achieving high accuracy and specificity. These results highlight its potential as a biomarker for early stroke diagnosis and as a foundation for real‐time, non‐invasive monitoring in clinical and prehospital settings.

## Graphical abstract

We developed an EEG‐based deep learning model to detect hyper‐acute large vessel occlusion stroke in mice. Using EEGNet, the model achieved > 95% accuracy across early post‐stroke intervals and reliably identified stroke within 1.5 h of onset. These findings suggest potential for real‐time, non‐invasive stroke triage.

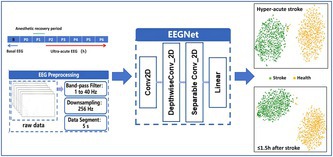

## 1 Introduction

Ischemic stroke is one of the leading causes of disability and death worldwide, particularly when it involves large vessel occlusion (LVO), where the severity is significantly higher [1, 2]. LVO often leads to extensive brain tissue damage, clinically manifesting as acute neurological deficits, frequently accompanied by severe impairments in motor, cognitive, and sensory functions [2, 3].

Endovascular thrombectomy (EVT) is the standard and most effective treatment for stroke caused by LVO [3, 4]. However, the efficacy of EVT is highly time‐dependent, and only a minority of hospitals are EVT‐capable [3, 5, 6]. As a result, stroke care systems must rapidly identify patients with LVO and transport them to EVT‐capable centers as quickly as possible. Unfortunately, current clinical assessments face challenges, including inconsistencies in diagnostic accuracy and comprehensiveness [7, 8]. While established modalities like computed tomography angiography, magnetic resonance angiography, and conventional angiography can provide reliable diagnoses, they are not feasible for prehospital use due to their time‐consuming nature and the need for specialized equipment [1, 9]. Thus, enhancing tools for early LVO detection, particularly in prehospital care, remains a crucial objective.

Electroencephalography (EEG) is a promising technique for prehospital stroke triage [10, 11, 12], which can immediately detect changes in brain function following the onset of brain ischemia, before cell death. Multiple studies have found moderate to high diagnostic accuracy of EEG for LVO detection [8, 13, 14]. However, the challenges of transforming EEG from a hospital‐based seizure diagnostic instrument into a prehospital stroke diagnostic are numerous.

One of the major challenges in utilizing EEG signals for clinical diagnosis is the efficient interpretation and automated recognition of the data [10]. Currently, EEG data analysis predominantly relies on manual labeling and traditional algorithms, which are not only time‐intensive but also require skilled professionals for accurate interpretation. Furthermore, existing studies are limited by the absence of EEG‐specific biomarkers capable of reliably assessing early‐stage stroke [15, 16, 17]. These limitations significantly hinder the practical application of EEG in the rapid assessment of acute stroke in emergency settings [16, 18]. Consequently, there is an urgent need for the development of more advanced algorithms to automate EEG data processing and accurately identify stroke‐specific features. By improving the performance of these algorithms, the diagnostic utility of EEG in clinical practice can be significantly enhanced, reducing human bias and enabling early, accurate detection of acute stroke. This, in turn, will provide stronger support for clinical decision‐making.

A further challenge in developing diagnostic algorithms is the necessity for a sufficiently large and reliable dataset [19]. In clinical settings, accurately capturing the time of stroke onset, particularly within the first 6 h, remains difficult [20]. Current research highlights a lack of diagnostic studies focusing specifically on this critical early time window [14]. To address this limitation, our study employs a permanent occlusion of the right middle cerebral artery (pMCAO) mouse model to simulate the stroke process [21] and obtain high‐resolution EEG data during the hyper‐acute phase. By leveraging deep learning techniques to extract pertinent features from these early EEG recordings, our goal is to establish a robust diagnostic framework capable of identifying stroke‐related biomarkers in the crucial hours after symptom onset, thereby providing valuable insights into early detection mechanisms and laying a foundational framework for future clinical investigations.

## 2 Methods

### 2.1 Animals

Twenty male mice of C57BL/6 (8–12 weeks old and weighing 25–30 g) were used in the study. Animals were maintained on a 12‐h light and dark cycle (light on at 8 AM) with freely available food and water. All animal experiments were reviewed and approved by the Institutional Animal Care and Use Committee (IACUC) of the Chinese Institute for Brain Research, Beijing, China (Approval ID: LARC‐T019). The procedures were deemed ethically acceptable and were conducted in accordance with the National Institutes of Health (NIH) Guide for the Care and Use of Laboratory Animals, the ARRIVE guidelines, and all relevant national regulations governing animal research.

### 2.2 Operation

#### 2.2.1 EEG Electrodes

**FIGURE 1:**
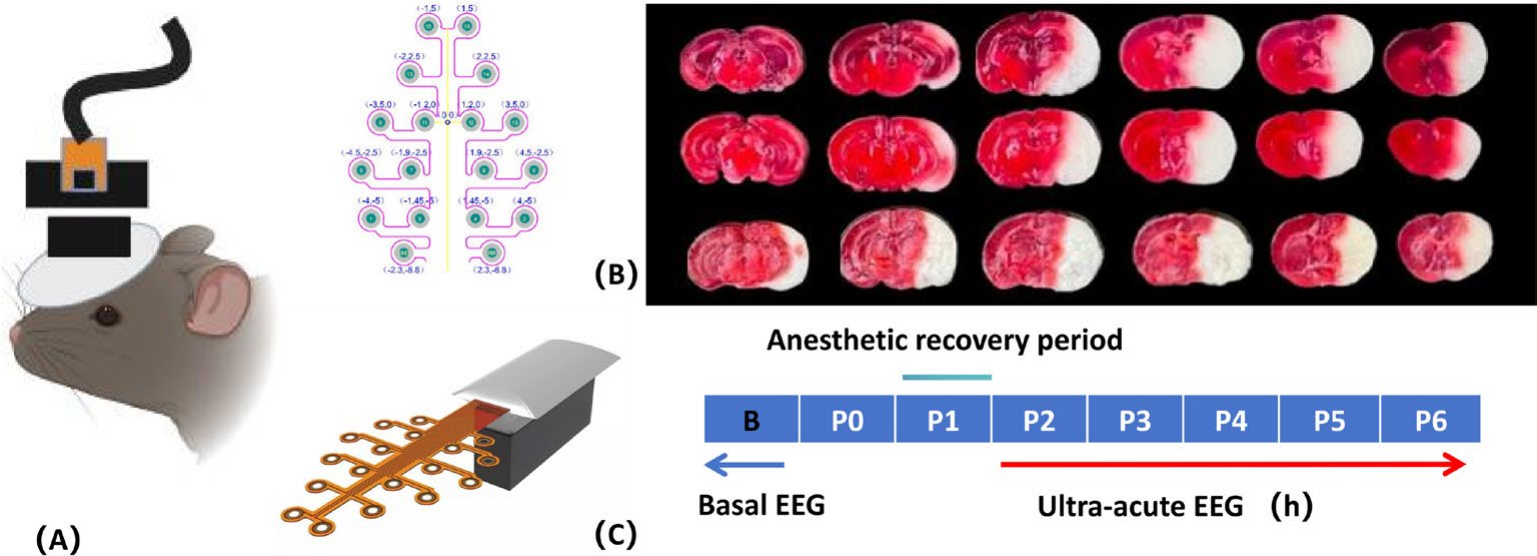
(A) Recording of EEG. Schematic illustration of the recording head‐stages on a mouse head, stainless steel 16 electrodes, and electrode coordinates relative to the bregma. (B) Hemispheric damage induced by permanent occlusion of the middle cerebral artery. (C) Experimental design for electroencephalography (EEG) recordings in mice. Following electrode implantation and a post‐surgical recovery period, mice were recorded over 7 consecutive days, 1 day baseline (pre‐stroke), 5 h poststroke (P2‐P6) and p'MCAO was induced at P0; P1 refers to the anesthesia recovery phase, during which EEG monitoring was not conducted.

Stainless steel 16 electrodes (Kedou Products) were employed to record electrical activity. Animals were anesthetized using isoflurane at a concentration of 3%, followed by 2% for maintenance during electrode placement. The recording electrodes were positioned in each hemisphere according to specific stereotaxic coordinates relative to the bregma, with the schematic diagram of electrode recording in Figure 1A. These electrodes were implanted subdurally through small holes drilled into the skull, secured with stainless steel screws (Kedou), and subsequently sealed with dental cement. Following the surgical procedure, mice received postoperative analgesia (Metachem, 0.1 mg/kg) and were allowed to recover for 7 days.

#### 2.2.2 pMCAO and Sham‐Operation

Ten mice received pMCAO. In brief, a silicon‐coated monofilament (Cinontech Ltd, Beijign, China) was introduced into the external carotid artery (ECA) and advanced carefully through the arterial lumen into the internal carotid artery (ICA), progressing toward the middle cerebral artery (MCA). The filament is advanced until a firm resistance is felt, indicating that it has reached the MCA and caused occlusion. Postoperatively, the mice are placed in a recovery chamber that is maintained at a warm temperature to prevent hypothermia. 5 mice received sham‐operation; sham‐operated animals underwent the same anesthesia and exposure of arteries without MCA occlusion [21, 22].

#### 2.2.3 TTC

Infarcts resulting from pMCAO were analyzed following treatment with 2,3,5‐triphenyltetrazolium chloride (TTC) 0.3 mice were euthanized through cervical dislocation 24 h post‐ischemic induction. The brains were carefully excised and sectioned into 1 mm thick coronal slices using a brain matrix (LEAGNE BIOTECHNOLOGY, DK0005). These slices were then stained with 1% TTC in 1% PBS at room temperature for 10 min. The stained coronal sections were digitized.

### 2.3 EEG Recording and Preprocessing

#### 2.3.1 EEG Recording

Prior to stroke, the mice underwent continuous EEG monitoring for a duration of 24 h. During the first hour post‐stroke, the mice were in the recovery phase from anesthesia, and no EEG monitoring was conducted. Subsequently, continuous EEG monitoring was performed for a duration of 5 h (Figure 1C). The EEG signals were acquired and analyzed using the KEDOUBC recording system (model RHD1024A, provided by Kedou (Suzhou) Brain‐Computer Technology Co. Ltd) at a 1 kHz sampling rate.

#### 2.3.2 EEG Preprocessing

EEG signals underwent preprocessing using MNE (v1.7.0), PyTorch (v2.2.2), NumPy (v1.24.3), SciPy (v1.10.1), and scikit‐learn (v1.4.2) with a 1–40 Hz band‐pass filter and down‐sampled to 256 Hz. Then, the EEG signals were divided into 5‐s segments, with each segment consisting of 1280 data points (5 s × 256 Hz). Each segment was labeled as either stroke (label = 0) or healthy (label = 1), and these segmented signals were used for training and testing the model.

### 2.4 Model Architecture and Evaluation

#### 2.4.1 *EEGNet* Model

**FIGURE 2:**
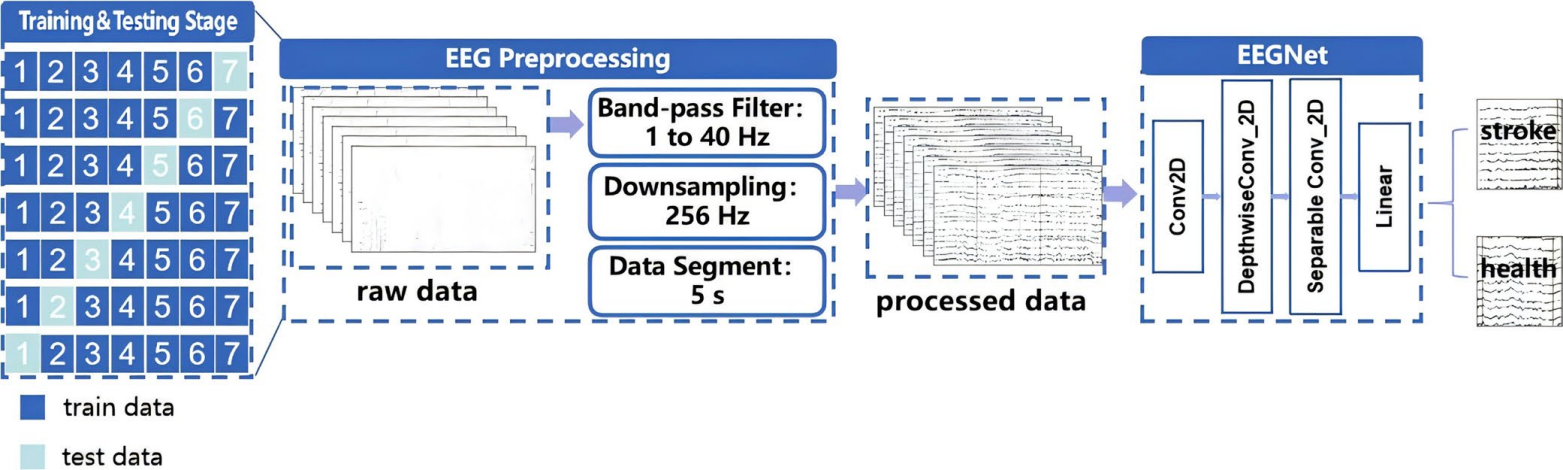
Research methodology flowchart. The diagram illustrates the sequential steps of the experimental pipeline. First, raw EEG data is acquired from mice during the hyper‐acute stroke phase. The data undergoes preprocessing, including band‐pass filtering (1–40 Hz), downsampling to 256 Hz, and segmentation into 5‐s epochs. These processed EEG segments are then used as input to the EEGNet model for binary classification (stroke vs. healthy). A 7‐fold cross‐validation strategy is applied to evaluate model performance. The integration of the preprocessing pipeline with the EEGNet architecture enables automated feature extraction and robust classification of stroke‐related EEG patterns.

In this experiment, we chose *EEGNet*, a lightweight convolutional neural network designed specifically for multichannel EEG signals. The model was configured with the default hyperparameters including a temporal filter length of F1 = 64, spatial‐depth multipliers D = 2, and dropout rate *p* = 0.5 [23]. The PyTorch implementation of *EEGNet* is publicly available at https://github.com/aliasvishnu/EEGNet. *EEGNet* extracts spatial and time‐frequency features via a series of convolutional operations. This architecture is well‐suited for processing high‐dimensional EEG signals, making it ideal for this study. Initially, the EEG data collected during the 5 h of the Hyper‐acute phase were fed into the *EEGNet* model for binary classification (stroke vs. healthy). A 7‐fold cross‐validation [24] was employed, where one mouse dataset was used as the test set and the remaining six as the training set. This process was repeated for each mouse, and the results were averaged across all seven experiments (Figure 2). Subsequently, EEG data were divided into six distinct time intervals for the same analysis: the first 30 min and consecutive hourly periods extending through the fifth hour. The models were implemented in PyTorch, running on an AMD CPU (R9‐3950X, 3.5 GHz) and an NVIDIA GPU (RTX 3090). The Adam optimizer with a learning rate of 0.001 was used to optimize the model's performance.

#### 2.4.2 Evaluation

Model performance was evaluated using the following metrics:

1. Accuracy (Acc): indicates the percentage of correct predictions of the model on the test set. This metric can help determine the overall performance of the model; TP (True Positives): True cases, which are predicted to be positive and are actually positive; FP (False Positives): False positives, which are predicted to be positive but are actually negative; FN (false Negatives): False Negatives, which are predicted to be negative but are actually positive; TN (True Negatives): True Negatives, which are predicted to be negative but are actually negative. The higher the value, the better the model's classification results.

$$\mathrm{Acc}=\frac{\mathrm{TP}+\mathrm{TN}}{\mathrm{TP}+\mathrm{FP}+\mathrm{FN}+\mathrm{TN}}$$

2. Precision: the proportion of correct predictions in samples where the prediction is positive, based on the prediction results. The higher the precision rate, the more reliable the results. Precision rate can be expressed by the formula.

$$\text{Precision}=\frac{\mathrm{TP}}{\mathrm{TP}+\mathrm{FP}}$$

3. Recall: the proportion of correctly predicted positive cases out of the total number of actual positive cases, based on the actual samples. The higher the recall, the fewer positive cases are missed by the model.

$$\text{Recall}=\frac{\mathrm{TP}}{\mathrm{TP}+\mathrm{FN}}$$

4. F1 Score: as a statistical metric to measure the balance between precision and recall of a binary classification model. A higher F1 score implies a higher precision and recall of the model, which is usually considered a sign of better model performance.

$$F1=2\times \frac{\text{Precision}\times \text{Recall}}{\text{Precision}+\text{Recall}}$$

5. ROC Curve and AUC: the Receiver Operating Characteristic (ROC) curve indicates the model's ability to discriminate between classes, with the area under the curve (AUC) providing a single value indicating performance. AUC values close to 1 indicate excellent discrimination.

6. Standard Deviation (std): standard Deviation is an important concept in statistics used to measure the degree of dispersion of results. A smaller standard deviation indicates a more centralized result, indicating a more stable model.

#### 2.4.3 Visualization of Classification Results (t‐SNE)

t‐Distributed Stochastic Neighbor Embedding (t‐SNE) [25] is a nonlinear dimensionality reduction technique used to visualize high‐dimensional data. It is particularly useful for revealing complex patterns in data. For this experiment, t‐SNE was applied to the output of the final layer of the *EEGNet* model to visualize the extracted features.

## 3 Results

In this study, 20 mice were used, of which 17 underwent electrode implantation, with 2 mice dying post‐surgery. Among the 15 mice that received successful electrode implantation, 10 underwent pMCAO surgery, 2 of which died within 6 h, and 1 was diagnosed with subarachnoid hemorrhage upon post‐mortem examination; 5 mice underwent sham surgery, with 1 dying post‐operation. Thus, the dataset for this experiment consisted of EEG recordings from 7 pMCAO mice and 4 sham‐operated mice. Moreover, 3 mice that underwent pMCAO surgery were euthanized 24 h later by cervical dislocation for TTC staining, with the infarct regions shown in Figure 1B.

### 3.1 Development of the Deep Learning Model Based on 5 h EEG Data

**FIGURE 3:**
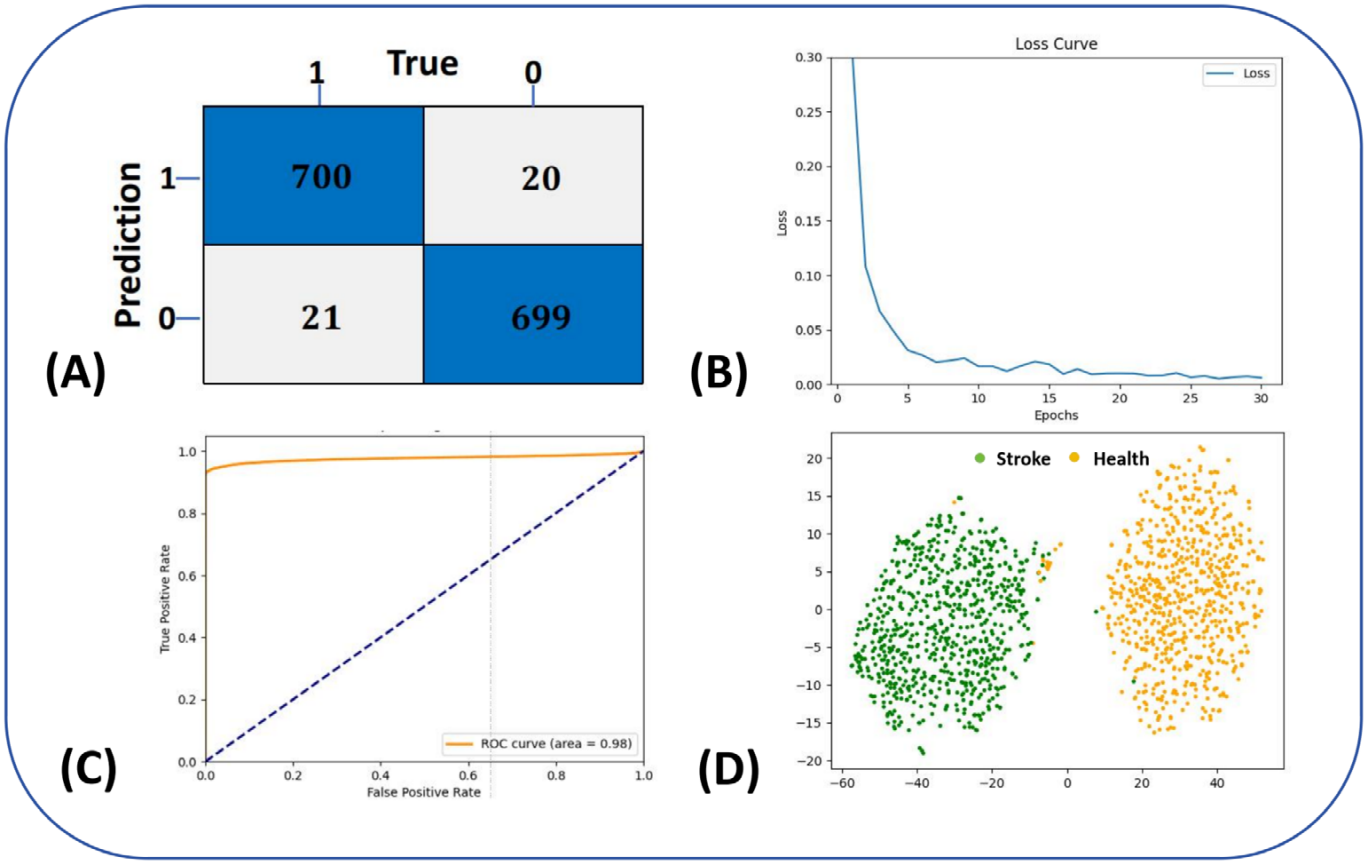
Development and evaluation of deep learning model based on 5 h hyperacute phase EEG. (A) Displays the confusion matrix constructed by the model, where 1 represents the positive class and 0 represents the negative class. (B) Loss curve, the horizontal axis is the number of iterations per epoch, and the vertical axis is the loss value. It can be seen that as the number of iterations per epoch increases, the loss value gradually decreases and becomes stable, indicating that the model is already in a convergence state. (C) ROC curve, the AUC is 0.98. (D) t‐SNE visualization of *EEGNet* features.

EEG features from mice during the entire hyperacute phase were extracted using the *EEGNet* algorithm, and a deep learning model was constructed based on these features. During the training process, the loss curve gradually decreased with an increasing number of epochs and eventually stabilized, indicating that the model had successfully converged. To further assess the stability and reliability of the model, we performed seven‐fold cross‐validation, evaluating the model's performance on different data subsets to ensure its generalizability and diagnostic accuracy. Through the comprehensive analysis of the entire hyperacute‐phase EEG data, we evaluated the model's performance using several key metrics, including ACC (0.979 ± 0.122), AUC (0.977 ± 0.031), precision (0.964 ± 0.124), recall (0.977 ± 0.175), and F1 score (0.966 ± 0.022). These metrics were used to assess the model's ability to differentiate between stroke and healthy states over the course of the hyperacute phase, providing a robust measure of the model's diagnostic performance and stability (Figure 3).

### 3.2 Evaluation of Deep Learning Model Across Hourly Intervals

To deepen our understanding of the dynamic changes in EEG signals following stroke, provide more accurate diagnostic insights, and enrich time‐series data for training deep learning models to enhance predictive performance, we segmented the hyper‐acute phase stroke model in mice into five distinct hourly intervals for analysis. The detailed evaluation parameters include ACC, AUC, precision, recall, F1 score, and Std for each time interval. As shown in Table 1, the model achieved high and stable accuracy throughout the 6‐h hyper‐acute phase, with values of 99.2% (within 1.5 h), 95.1% (2 h), 98.0% (3 h), 99.8% (4 h), 97.1% (5 h), and 97.0% (6 h). Similarly, AUC, precision, recall, and F1 scores remained above 0.95 across nearly all intervals, with only minor fluctuations observed (e.g., a modest dip in F1 score at 3 h to 0.936). These variations are likely due to physiological variability or sample‐level randomness and do not indicate a systematic degradation in performance. Given the uniformly strong classification results, the differences between time intervals are relatively small and may hold limited statistical or practical significance. This further supports the model's consistent performance and diagnostic utility throughout the early post‐stroke period.

**TABLE 1:** Evaluation metrics of deep learning models across different classifications.

Classification	Acc ± std	Auc ± std	Precision ± std	Recall ± std	F1 ± std
Within 1.5 h	0.992 ± 0.013	0.982 ± 0.023	0.993 ± 0.012	0.992 ± 0.013	0.982 ± 0.017
2 h	0.951 ± 0.031	0.986 ± 0.017	0.961 ± 0.021	0.951 ± 0.031	0.951 ± 0.031
3 h	0.980 ± 0.038	0.988 ± 0.021	0.978 ± 0.022	0.980 ± 0.038	0.936 ± 0.036
4 h	0.998 ± 0.011	0.991 ± 0.034	0.996 ± 0.078	0.984 ± 0.011	0.988 ± 0.010
5 h	0.971 ± 0.039	0.972 ± 0.019	0.976 ± 0.029	0.974 ± 0.039	0.967 ± 0.037
6 h	0.970 ± 0.120	0.986 ± 0.022	0.978 ± 0.019	0.971 ± 0.025	0.974 ± 0.025
Sham operation	0.973 ± 0.012	0.972 ± 0.009	0.966 ± 0.022	0.964 ± 0.028	0.974 ± 0.016

Abbreviations: Acc, accuracy; AUC, the area under the curve; std., standard deviation.

### 3.3 Early Diagnostic Capability of the Deep Learning Model

**FIGURE 4:**
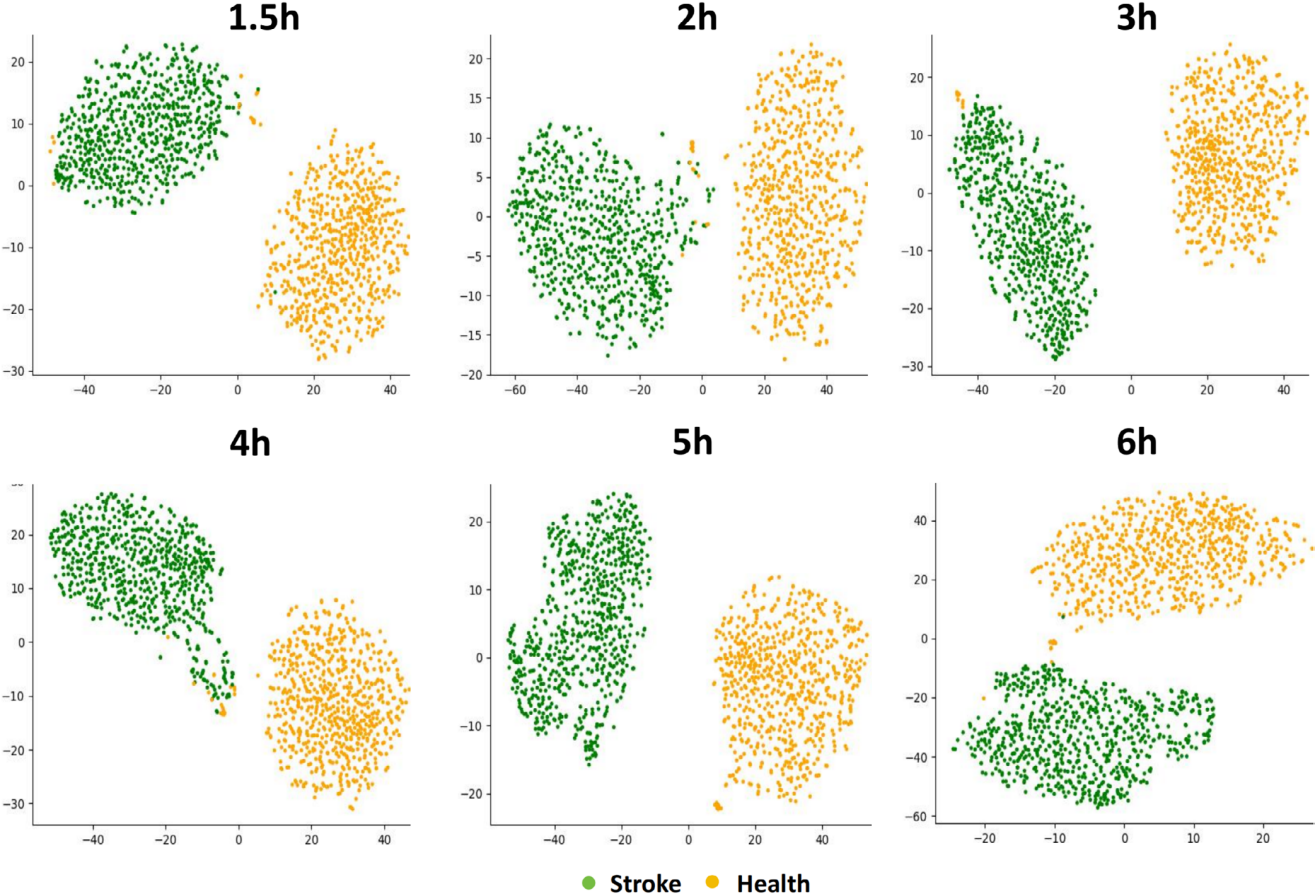
t‐SNE results across different time segments, the results demonstrate that the deep learning model can accurately distinguish different events within each time segment.

Given that the model demonstrated high accuracy in distinguishing stroke and healthy states across various time periods, particularly during the 1–22 h, we further investigated the earliest time point at which the model could reliably detect stroke. To this end, we analyzed EEG data collected within the first 1.5 h following stroke onset. As shown in Table 1 and Figure 4A, which present the classification metrics and t‐SNE visualization respectively, the model maintained exceptionally high classification accuracy during the early period, with an accuracy of 99.2% (0.992 ± 0.013) and an AUC of 0.982 ± 0.023. These findings suggest that the model retains strong diagnostic capability even in the hyper‐acute phase, within the first 1.5 h after stroke onset.

### 3.4 Distinguishing Stroke From Sham Stroke Using Deep Learning Model

To eliminate the potential influence of anesthesia or other confounding factors on the EEG data, we compared the EEG recordings from the pMCAO group and the sham group. As shown in Table 1, the results demonstrated that the classification model was able to accurately distinguish between the two groups. This finding suggests that the excellent performance of the model is closely associated with the occurrence of stroke rather than being attributed to anesthesia or other external factors. By analyzing the significant differences between the pMCAO and sham groups, we further validated the specific changes in EEG signals associated with the stroke state. These findings confirm that the classification model exhibits high accuracy and reliability in differentiating between stroke and non‐stroke conditions.

### 3.5 T‐SNE Analysis of Model Performance in Stroke Differentiation

Based on t‐SNE analysis, the results demonstrate that the deep learning model can accurately distinguish different events within each time segment we defined. By mapping the EEG signals of each time interval into a lower‐dimensional space, we observed significant signal separation, indicating that the model effectively extracted key features differentiating stroke from healthy states (Figure 4). Notably, the model was able to independently and accurately distinguish events in each time segment, showcasing its superior performance and stability in handling EEG time‐series data. This finding further confirms the model's capability to address the rapidly changing dynamic features of hyperacute stroke and provides strong support for early stroke diagnosis.

## 4 Discussion

In this study, we employed the *EEGNet* deep learning framework to identify the hyper‐acute phase of ischemic stroke in a mouse model induced by anterior circulation occlusion. Our results demonstrated that EEG data holds significant diagnostic value in the identification of hyper‐acute stroke. The model based on EEG data achieved robust classification performance, effectively distinguishing LVO stroke across all analyzed time intervals within the hyper‐acute phase. Notably, the earliest time point where the model achieved successful identification was 1–1.55 h post‐stroke induction.

### 4.1 Deep Learning Model as Potential Biomarker for Hyper‐Acute LVO Stroke Detection

LVO stroke represents one of the most severe subtypes of acute ischemic stroke, often associated with high mortality and disability rates. Early and accurate identification of LVO stroke is crucial for timely intervention, as the thrombectomy window for anterior circulation is typically only 6 h [3, 26]. EEG has the potential for early stroke detection and shows high prospects for application in acute stroke diagnosis [18, 27]. Several clinical studies have explored the changes in EEG following LVO stroke, obtaining statistically significant results. Shreve et al. found delta band power (*p* = 0.004) and the alpha/delta frequency band ratio (*p* = 0.0006) each significantly distinguished patients with large acute ischemic stroke from all other patients with suspected stroke [28]. The study by van Meenen LCC et al. identified alterations in theta and alpha power; a combination of the weighted phase lag index and relative theta power provided the most accurate diagnostic performance, achieving a sensitivity of 100% and a specificity of 84% [18]. Fareshte demonstrated that combining four Lasso‐selected EEG lead‐band pairs, including alpha, low beta, and high beta bands, with clinical information in deep learning models can achieve high diagnostic accuracy, with an AUC of 0.86, sensitivity of 76%, and specificity of 80% [8]. These results suggest that EEG may be useful to improve the diagnosis of large acute ischemic stroke, findings that might be useful for pre‐hospital applications.

However, few diagnostic studies to date have exclusively focused on patients within the critical 6 h window after symptom onset, a period that is pivotal for effective treatment of LVO stroke [13, 14, 28]. Additionally, while there are numerous reports from animal studies on EEG changes following acute stroke, most focus on spectral changes; different studies lack standardized metrics for spectral changes, which diminishes their specificity and diagnostic value [12, 29, 30, 31]. In this study, we utilized the pMCAO mouse model to simulate LVO stroke and employed deep learning methods to confirm the significant value of EEG signals in identifying hyper‐acute stroke. This study clearly confirms that EEG data holds significant diagnostic value in the identification of hyper‐acute stroke. The deep learning model based on EEG data showed excellent recognition capability, with classification accuracy consistently above 95% across all time intervals. The AUC values approaching 1 demonstrated the model's strong consistency and reliable performance in distinguishing stroke from healthy states. Additionally, the small standard deviations indicated stable and robust predictions across different time periods. These evaluation metrics and excellent performance suggest that the EEG‐based deep learning model has the potential to serve as a biomarker for identifying LVO stroke. In this study, EEG data from the first hour following stroke onset were not recorded, as this period overlaps with the recovery phase from anesthesia. To minimize potential interference from anesthesia‐induced alterations in cortical activity, EEG acquisition was initiated only after the animals resumed stable, spontaneous behavior. This approach ensured that the recorded signals reflected authentic post‐stroke neural dynamics.

Early diagnosis of stroke is critical for improving treatment outcomes, as therapeutic efficacy is highly time‐dependent [32]. Notably, even without access to data from the earliest time window (0–1 h), the model demonstrated excellent classification performance within the 1–1.55 h interval, achieving high accuracy and discriminability despite the expected subtlety of EEG changes at such an early stage. This result suggests that the model is capable of detecting ischemic events shortly after onset. Future work may aim to collect EEG data during the first hour post‐stroke to determine whether detection can be pushed even earlier, potentially enhancing clinical decision‐making in ultra‐early stroke care.

### 4.2 Advanced Algorithms Enhance EEG‐Based Stroke Detection With More Simplicity and Specificity

Previous applications of EEG in acute ischemic stroke have primarily focused on analyzing spectral power and the Brain Symmetry Index (BSI) [12]. These studies typically require significant time and effort for analysis, necessitating specialized expertise for analysis and interpretation. Moreover, the results lack sufficient specificity, with discrepancies observed across different studies, making it challenging to establish a unified standard or diagnostic marker. This is also the primary reason why EEG data is challenging to use in acute stroke diagnosis. In this study, we applied the *EEGNet* deep learning algorithm to construct a diagnostic model based on hyper‐acute mouse EEG data. Compared to traditional diagnostic methods, this model not only has the capability to capture subtle and complex signal variations while extracting high‐dimensional latent features from temporal data, but also streamlines the processing workflow, allowing for results to be obtained simply by inputting EEG data, thereby reducing the need for specialized knowledge in EEG analysis [23, 33, 34]. This ability opens new avenues for dynamic monitoring and classification of stroke states, laying a foundation for the future development of non‐invasive, real‐time stroke diagnostic tools with significant clinical utility. Specifically, the proposed EEGNet‐based model demonstrates strong translational potential. EEG is a non‐invasive, widely available neuroimaging modality, and its portability makes it particularly well‐suited for prehospital stroke screening [7, 17, 18]. The compact architecture and low computational latency of EEGNet further support its integration into edge devices or portable EEG systems for use in emergency scenarios. Although our current study is based on the pMCAO mouse model, EEG biomarkers of cerebral ischemia, such as focal slowing and spectral power alterations, are well‐established in early human stroke, offering a physiological foundation for translational application [12]. Future work will focus on adapting and validating the model using human EEG datasets collected during the hyper‐acute phase of stroke in both clinical and prehospital settings. Furthermore, with ongoing advancements in portable EEG technologies and wearable brain‐monitoring systems, it is now feasible to acquire EEG data in mobile or ambulance‐based environments. Given EEGNet's lightweight structure and compatibility with low‐power hardware, the proposed model can be seamlessly integrated into real‐time processing pipelines for stroke triage [23, 33]. Such integration could facilitate earlier diagnosis and transport decisions, ultimately reducing treatment delays and improving patient outcomes.

### 4.3 Cross‐Validation and Sham Comparison for Reliable Model Identification

Given the relatively small sample size in this study, we implemented 7‐fold cross‐validation to ensure the reliability of the experimental results and the generalizability of the model. In the 7‐fold cross‐validation process, the stroke dataset was randomly divided into seven subsets. Six of these subsets were used for model training, while the remaining one was used for testing. This process was repeated seven times, ensuring that each sample was included in both the training and testing phases. This approach not only minimizes the bias introduced by the small sample size but also helps prevent overfitting, thereby enhancing the model's adaptability and stability in practical applications [35]. Additionally, to eliminate the potential confounding effects of anesthesia or other extraneous factors that might influence EEG signals, we compared the EEG data from the pMCAO stroke model with that of a sham stroke group. This comparative analysis ensured that the EEG alterations observed in the stroke group were indeed due to the stroke itself, rather than being influenced by non‐pathological factors such as anesthesia recovery or surgical procedures. The inclusion of the sham‐operated group provided further validation for the specificity of the EEG features observed in the ischemic stroke model, reinforcing the robustness and relevance of our findings in identifying stroke‐related EEG biomarkers.

### 4.4 Limitations and Future Directions

In this study, although we integrated optimized algorithms with EEG data to achieve robust results, it is important to acknowledge that the research remains in exploratory stages, there are several limitations. First, one major limitation of this study is the relatively small sample size, consisting of seven pMCAO and four sham‐operated mice. While we employed 7‐fold cross‐validation to mitigate the effects of overfitting and improve the reliability of the model evaluation, we acknowledge that the statistical power remains limited. A small dataset may reduce the generalizability of the findings and increase the risk of model bias, particularly in detecting subtle signal variations. Future research should include larger and more diverse animal cohorts to further validate the model's robustness and stability. Another important limitation is the inherent physiological and pathological differences between mice and humans, which may affect the translational relevance of the findings. Although the pMCAO mouse model provides a well‐established and controlled platform for simulating LVO stroke and collecting high‐resolution EEG data, it cannot fully replicate the complexity of human stroke pathophysiology. Therefore, caution must be exercised when applying these results to human populations. Future studies should include clinical validation using EEG recordings from stroke patients to assess whether the proposed model performs similarly in real‐world clinical environments. Future research could expand in several directions. On the one hand, investigations should include other stroke subtypes, such as ischemic and hemorrhagic strokes, to evaluate the broader applicability of deep learning models. On the other hand, advancements in EEG acquisition technology and deep learning algorithms promise higher‐resolution data and more sophisticated signal processing capabilities. These improvements may enable real‐time analysis and one‐click identification of EEG data, facilitating the use of EEG as a practical tool for pre‐hospital acute stroke diagnosis. Such developments could significantly reduce diagnostic delays and improve outcomes for patients by providing efficient and accurate solutions for hyper‐acute stroke detection [36].

## 5 Conclusion

In conclusion, this study further confirms the potential of EEG in hyper‐acute stroke diagnosis, laying a theoretical foundation for the future development of non‐invasive, real‐time stroke monitoring tools. Using a pMCAO mouse model, the research successfully identified LVO stroke states during the hyper‐acute phase with high accuracy. The deep learning framework demonstrated exceptional sensitivity to subtle EEG variations, enabling precise and reliable classification. Although the study is limited by a small sample size and its focus on LVO strokes, the findings provide valuable insights into the feasibility of EEG‐based stroke detection. With advancements in algorithms and real‐time EEG technologies, the prospect of one‐click stroke diagnosis in prehospital settings holds significant promise for improving stroke care.

## Author Contributions

Tan Zhang, Xiaolin Li contributed to the study methodology, software, formal analysis, visualization, and writing original draft. Xinxin Hu contributed to the study methodology, software, formal analysis, and visualization. Qingchun Mu, Xiaoke Chai, and Zhiyong Zhou contributed to the study conceptualization, formal analysis, and writing review and editing. Qing Lan and Jizong Zhao supervised, carried out project administration, writing review and editing, and acquired funding.

## Ethics Statement

All animal experimental procedures were approved by the ethics committee of the Chinese Institute for Brain Research (Approval ID: LARC‐T019). The study complied with the ARRIVE guidelines and relevant national regulations for the use of animals in scientific research. This article did not contain any studies with human participants.

## Conflicts of Interest

The authors declare no conflicts of interest.

## Data Availability

The datasets generated and analyzed during the current study are available from the corresponding author upon reasonable request.

## References

[1] N. A. Hilkens , B. Casolla , T. W. Leung , and F. E. de Leeuw , “Stroke,” Lancet 403, no. 10446 (2024): 2820–2836. 38759664 10.1016/S0140-6736(24)00642-1

[2] L. A. Beume , M. Hieber , C. P. Kaller , et al., “Large Vessel Occlusion in Acute Stroke,” Stroke 49, no. 10 (2018): 2323–2329. 30355088 10.1161/STROKEAHA.118.022253

[3] T. N. Nguyen , M. Abdalkader , U. Fischer , et al., “Endovascular Management of Acute Stroke,” Lancet 404, no. 10459 (2024): 1265–1278. 39341645 10.1016/S0140-6736(24)01410-7

[4] H. Chen , J. S. Lee , P. Michel , B. Yan , and S. Chaturvedi , “Endovascular Stroke Thrombectomy for Patients With Large Ischemic Core: A Review,” JAMA Neurology 81, no. 10 (2024): 1085–1093. 39133467 10.1001/jamaneurol.2024.2500

[5] E. E. Smith , D. M. Kent , K. R. Bulsara , et al., “Accuracy of Prediction Instruments for Diagnosing Large Vessel Occlusion in Individuals With Suspected Stroke: A Systematic Review for the 2018 Guidelines for the Early Management of Patients With Acute Ischemic Stroke,” Stroke 49, no. 3 (2018): e111–e122. 29367333 10.1161/STR.0000000000000160

[6] N. Arrarte Terreros , A. Bruggeman , I. Swijnenburg , et al., “Early Recanalization in Large‐Vessel Occlusion Stroke Patients Transferred for Endovascular Treatment,” Journal of Neurointerventional Surgery 14, no. 5 (2022): 480–484. 33986112 10.1136/neurintsurg-2021-017441 PMC9016237

[7] A. de Havenon , I. Ayodele , B. Alhanti , et al., “Prediction of Large Vessel Occlusion Stroke Using Clinical Registries for Research,” Neurology 102, no. 11 (2024): e209424. 38759133 10.1212/WNL.0000000000209424 PMC11175650

[8] F. Erani , N. Zolotova , B. Vanderschelden , et al., “Electroencephalography Might Improve Diagnosis of Acute Stroke and Large Vessel Occlusion,” Stroke 51, no. 11 (2020): 3361–3365. 32942967 10.1161/STROKEAHA.120.030150 PMC7606743

[9] P. Seners , J. C. Baron , J. M. Olivot , and G. W. Albers , “Does Imaging of the Ischemic Penumbra Have Value in Acute Ischemic Stroke With Large Vessel Occlusion,” Current Opinion in Neurology 37, no. 1 (2024): 1–7. 38038427 10.1097/WCO.0000000000001235

[10] N. Sanossian and E. Fink , “What Will the Mobile Stroke Unit of the Future Look Like, and Will EEG Have a Role,” Neurology 101, no. 24 (2023): 1085–1086. 37848337 10.1212/WNL.0000000000208047

[11] P. García‐Peña , M. Ramos , J. M. López , R. Martinez‐Murillo , G. de Arcas , and D. Gonzalez‐Nieto , “Preclinical Examination of Early‐Onset Thalamic‐Cortical Seizures After Hemispheric Stroke,” Epilepsia 64, no. 9 (2023): 2499–2514. 37277947 10.1111/epi.17675

[12] Y. Sato , O. Schmitt , Z. Ip , et al., “Pathological Changes of Brain Oscillations Following Ischemic Stroke,” Journal of Cerebral Blood Flow and Metabolism 42, no. 10 (2022): 1753–1776. 35754347 10.1177/0271678X221105677 PMC9536122

[13] E. A. Groenendijk , M. N. van Stigt , A. A. G. A. van de Munckhof , et al., “Subhairline Electroencephalography for the Detection of Large Vessel Occlusion Stroke,” Journal of the American Heart Association 12, no. 22 (2023): e031929. 37982212 10.1161/JAHA.123.031929 PMC10727307

[14] L. Sutcliffe , H. Lumley , L. Shaw , R. Francis , and C. I. Price , “Surface Electroencephalography (EEG) During the Acute Phase of Stroke to Assist With Diagnosis and Prediction of Prognosis: A Scoping Review,” BMC Emergency Medicine 22, no. 1 (2022): 29. 35227206 10.1186/s12873-022-00585-w PMC8883639

[15] L. van Meenen , M. N. van Stigt , A. Siegers , et al., “Detection of Large Vessel Occlusion Stroke in the Prehospital Setting: Electroencephalography as a Potential Triage Instrument,” Stroke 52, no. 7 (2021): e347–e355. 33940955 10.1161/STROKEAHA.120.033053

[16] M. N. van Stigt , E. A. Groenendijk , L. C. C. van Meenen , et al., “Prehospital Detection of Large Vessel Occlusion Stroke With EEG,” Neurology 101, no. 24 (2023): e2522–e2532. 37848336 10.1212/WNL.0000000000207831 PMC10791060

[17] K. B. Walsh , “Non‐Invasive Sensor Technology for Prehospital Stroke Diagnosis: Current Status and Future Directions,” International Journal of Stroke 14, no. 6 (2019): 592–602. 31354081 10.1177/1747493019866621

[18] L. C. C. van Meenen , M. N. van Stigt , A. Siegers , et al., “Detection of Large Vessel Occlusion Stroke With Electroencephalography in the Emergency Room: First Results of the ELECTRA‐STROKE Study,” Journal of Neurology 269, no. 4 (2022): 2030–2038. 34476587 10.1007/s00415-021-10781-6 PMC8412867

[19] M. Peycheva , A. Seiler , F. Wagner , L. Li , and M. R. Heldner , “Journal Club: Prehospital Detection of Large Vessel Occlusion Stroke With Electroencephalography: Results of the ELECTRA‐STROKE Study,” Neurology 103, no. 2 (2024): e209587. 38870459 10.1212/WNL.0000000000209587 PMC12296629

[20] C. Z. Simonsen , T. M. Leslie‐Mazwi , and G. Thomalla , “Which Imaging Approach Should be Used for Stroke of Unknown Time of Onset,” Stroke 52, no. 1 (2021): 373–380. 33302796 10.1161/STROKEAHA.120.032020

[21] S. K. Narayan , S. Grace Cherian , P. Babu Phaniti , S. Babu Chidambaram , A. H. Rachel Vasanthi , and M. Arumugam , “Preclinical Animal Studies in Ischemic Stroke: Challenges and Some Solutions,” Animal Models and Experimental Medicine 4, no. 2 (2021): 104–115. 34179718 10.1002/ame2.12166 PMC8212819

[22] T. Chiang , R. O. Messing , and W. H. Chou , “Mouse Model of Middle Cerebral Artery Occlusion,” Journal of Visualized Experiments 48 (2011): 2761. 10.3791/2761 PMC3197421 21372780

[23] V. J. Lawhern , A. J. Solon , N. R. Waytowich , S. M. Gordon , C. P. Hung , and B. J. Lance , “EEGNet: A Compact Convolutional Neural Network for EEG‐Based Brain‐Computer Interfaces,” Journal of Neural Engineering 15, no. 5 (2018): 056013. 29932424 10.1088/1741-2552/aace8c

[24] H. Chen , Y. Wang , T. Ji , Y. Jiang , and X. H. Zhou , “Brain Functional Connectivity‐Based Prediction of Vagus Nerve Stimulation Efficacy in Pediatric Pharmacoresistant Epilepsy,” CNS Neuroscience & Therapeutics 29, no. 11 (2023): 3259–3268. 37170486 10.1111/cns.14257 PMC10580342

[25] X. Xu , Z. Xie , Z. Yang , D. Li , and X. Xu , “A t‐SNE Based Classification Approach to Compositional Microbiome Data,” Frontiers in Genetics 11 (2020): 620143. 33381156 10.3389/fgene.2020.620143 PMC7767995

[26] V. Costalat , B. Lapergue , J. F. Albucher , et al., “Evaluation of Acute Mechanical Revascularization in Large Stroke (ASPECTS ⩽5) and Large Vessel Occlusion Within 7 h of Last‐Seen‐Well: The LASTE Multicenter, Randomized, Clinical Trial Protocol,” International Journal of Stroke 19, no. 1 (2024): 114–119. 37462028 10.1177/17474930231191033

[27] Y. Yan , X. An , Y. Ma , et al., “Detection of Early Neurological Deterioration Using a Quantitative Electroencephalography System in Patients With Large Vessel Occlusion Stroke After Endovascular Treatment,” Journal of Neurointerventional Surgery 17 (2024): 883–889. 10.1136/jnis-2024-022011 PMC12322402 39053935

[28] L. Shreve , A. Kaur , C. Vo , et al., “Electroencephalography Measures Are Useful for Identifying Large Acute Ischemic Stroke in the Emergency Department,” Journal of Stroke and Cerebrovascular Diseases 28, no. 8 (2019): 2280–2286. 31174955 10.1016/j.jstrokecerebrovasdis.2019.05.019 PMC6790298

[29] D. J. Griggs , K. Khateeb , J. Zhou , T. Liu , R. Wang , and A. Yazdan‐Shahmorad , “Multi‐Modal Artificial Dura for Simultaneous Large‐Scale Optical Access and Large‐Scale Electrophysiology in Non‐Human Primate Cortex,” Journal of Neural Engineering 18, no. 5 (2021): 55006. 10.1088/1741-2552/abf28d PMC8523212 33770770

[30] A. Yazdan‐Shahmorad , C. Diaz‐Botia , T. L. Hanson , et al., “A Large‐Scale Interface for Optogenetic Stimulation and Recording in Nonhuman Primates,” Neuron 89, no. 5 (2016): 927–939. 26875625 10.1016/j.neuron.2016.01.013

[31] S. J. Zhang , Z. Ke , L. Li , S. P. Yip , and K. Y. Tong , “EEG Patterns From Acute to Chronic Stroke Phases in Focal Cerebral Ischemic Rats: Correlations With Functional Recovery,” Physiological Measurement 34, no. 4 (2013): 423–435. 23524534 10.1088/0967-3334/34/4/423

[32] X. Huo , A. Jin , Z. Miao , Y. Wang , and D. Wang , “When Treating Acute Ischaemic Stroke of LVO Type, Time Window Prevails Over Tissue Window,” Stroke and Vascular Neurology 9, no. 5 (2024): 461–463. 38164603 10.1136/svn-2023-003007 PMC11732835

[33] R. Kessler , A. Enge , and M. A. Skeide , “How EEG Preprocessing Shapes Decoding Performance,” Communications Biology 8, no. 1 (2025): 1039. 40640472 10.1038/s42003-025-08464-3 PMC12246244

[34] X. Zhang , L. Xie , W. Liu , et al., “Exoskeleton‐Guided Passive Movement Elicits Standardized EEG Patterns for Generalizable BCIs in Stroke Rehabilitation,” Journal of NeuroEngineering and Rehabilitation 22, no. 1 (2025): 97. 40287725 10.1186/s12984-025-01627-7 PMC12032773

[35] S. Bates , T. Hastie , and R. Tibshirani , “Cross‐Validation: What Does It Estimate and How Well Does It Do It,” Journal of the American Statistical Association 119, no. 546 (2024): 1434–1445. 39308484 10.1080/01621459.2023.2197686 PMC11412612

[36] J. C. Grotta , J. M. Yamal , S. A. Parker , et al., “Prospective, Multicenter, Controlled Trial of Mobile Stroke Units,” New England Journal of Medicine 385, no. 11 (2021): 971–981. 34496173 10.1056/NEJMoa2103879

